# Inhibition of SARS-CoV-2 Entry into Host Cells Using Small Molecules

**DOI:** 10.3390/ph13120447

**Published:** 2020-12-08

**Authors:** Kenana Al Adem, Aya Shanti, Cesare Stefanini, Sungmun Lee

**Affiliations:** 1 Healthcare Engineering Innovation Center, Department of Biomedical Engineering, Khalifa University of Science and Technology, Abu Dhabi 127788, UAE; kenana.aladem@ku.ac.ae (K.A.A.); aya.shanti@ku.ac.ae (A.S.); cesare.stefanini@ku.ac.ae (C.S.); 2 Khalifa University’s Center for Biotechnology, Khalifa University of Science and Technology, Abu Dhabi 127788, UAE

**Keywords:** Covid-19, SARS-CoV-2, spike protein, angiotensin converting enzyme 2, small molecule, inhibitors

## Abstract

Severe Acute Respiratory Syndrome Coronavirus 2 (SARS-CoV-2), a virus belonging to the Coronavirus family, is now known to cause Coronavirus Disease (Covid-19) which was first recognized in December 2019. Covid-19 leads to respiratory illnesses ranging from mild infections to pneumonia and lung failure. Strikingly, within a few months of its first report, Covid-19 has spread worldwide at an exceptionally high speed and it has caused enormous human casualties. As yet, there is no specific treatment for Covid-19. Designing inhibitory drugs that can interfere with the viral entry process constitutes one of the main preventative therapies that could combat SARS-CoV-2 infection at an early stage. In this review, we provide a brief introduction of the main features of coronaviruses, discuss the entering mechanism of SARS-CoV-2 into human host cells and review small molecules that inhibit SARS-CoV-2 entry into host cells. Specifically, we focus on small molecules, identified by experimental validation and/or computational prediction, that target the SARS-CoV-2 spike protein, human angiotensin converting enzyme 2 (ACE2) receptor and the different host cell proteases that activate viral fusion. Given the persistent rise in Covid-19 cases to date, efforts should be directed towards validating the therapeutic effectiveness of these identified small molecule inhibitors.

## 1. Introduction

Viral diseases constitute a serious global health issue [1]. In just the last twenty years, the human population has faced several viral epidemics including the Severe Acute Respiratory Syndrome (SARS) in 2002–2003, the Swine Flu in 2009, the Middle East Respiratory Syndrome (MERS) in 2012 and the most recent Coronavirus Disease (Covid-19) first identified in late 2019. Covid-19 is still spreading at an extremely high pace across the globe. To date, it has infected more than 44 million people worldwide and claimed the lives of more than a million people. Up to the current time, there are no specific treatments or vaccines approved for this disease. Some countries have authorized the use of already existing antiviral drugs such as remdesivir and favipiravir for the treatment of Covid-19 in an effort to combat the disease. However, conclusive results on whether these medications are effective or not are still lacking.

Covid-19 is caused by a virus now known as the Severe Acute Respiratory Syndrome Coronavirus 2 (SARS-CoV-2) belonging to the same family of viruses that caused the SARS and MERS diseases, called the Coronavirus family. In fact, the constant outbreaks of coronaviruses signify that these viruses represent a serious threat to the human population and emphasize the need to develop effective treatment modalities against them as soon as possible. In this review, we summarize the main features of coronaviruses, discuss the entering mechanism of SARS-CoV-2 into human host cells and review small molecules that specifically inhibit SARS-CoV-2 entry into host cells. 

### 1.1. Classification of Coronaviruses

Coronaviruses (CoVs) are the largest family of viruses belonging to the order Nidovirales, which includes three other families, namely Mesoniviridae, Roniviridae, and Arteriviridae [2,3]. Members of this order share several distinctive characteristics including (1) they are surrounded by an envelope, (2) they contain very large genomes (≥30 kilobases), (3) they have a highly conserved genomic organization, (4) they express many nonstructural genes and (5) they replicate using a nested set of mRNAs, from which the name of the order is derived (nido is Latin for the word “nest”) [2]. The differences between the members of the Nidovirales order are due to differences in the number, type and size of the structural proteins they express [2].

The CoV family is further divided into four genera: alpha, beta, gamma and delta CoVs. Genomic characterizations demonstrate that rodents and bats are the most probable gene sources of the alpha and beta CoVs while the avian species are the most probable gene sources of the gamma and delta CoVs [1]. All pathogenic human CoVs belong to either alpha or beta CoVs. They usually infect the upper respiratory tract, but they have been shown to also infect the gastrointestinal tract and central nervous system in some cases [3,4,5,6,7,8,9].

### 1.2. General Structure of Coronaviruses

CoVs are spherical viruses with diameters of approximately 125 nm [10,11]. They are characterized by club-shaped spike projections protruding from their surfaces, which under the electron microscope, create an image similar to a solar corona (corona is Latin for the word “crown”) [2]. They contain a single stranded, nonsegmented, positive-sense RNA genome that has a 5′ cap and a 3′ poly (A) tail which enable it to act as a messenger RNA for translation of replicase polyproteins once inside the host cell [2]. The genome of CoVs encodes nonstructural proteins, structural proteins and accessory proteins. The genes encoding nonstructural proteins occupy two thirds of the genome while genes encoding structural and accessory proteins encode only one third of the genome.

Nonstructural proteins are essential for directing RNA synthesis and processing, for cellular mRNA degradation, for host immune response suppression and for double membrane vesicle formation [12,13,14,15,16]. Structural proteins, on the other hand, are essential for the protection of the viral genome, for the infection and entry of the virus into host cells, for the assembly of new viral particles and for the export of new viral particles out of the host cells [17,18,19,20]. Lastly, accessory proteins are mainly involved in viral pathogenesis [21,22].

Genome sequence alignment of CoVs demonstrates that nonstructural protein coding regions are more conserved than structural protein coding regions with 58% similarity in nonstructural protein coding regions versus 43% similarity in structural protein coding regions across different CoVs [3]. This indicates that structural proteins might be in need of constant evolution in order to adapt to new hosts.

## 2. Structural Proteins of Coronaviruses

**Figure 1:**
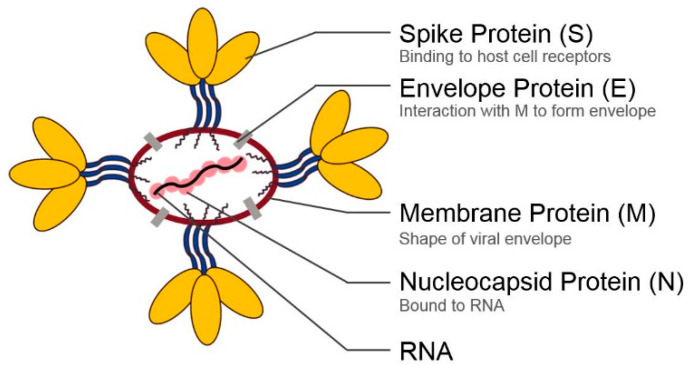
Basic structure of SARS-CoV-2 including its genome RNA and the four main structural proteins. The schematic is not drawn to scale. Enlargement has been employed to better depict the structures.

CoVs contain four main structural proteins, namely nucleocapsid (N) protein, membrane (M) protein, envelope (E) protein and spike (S) protein as shown in Figure 1. The N protein is a 45 kDa RNA-binding protein composed of two separate domains, an N-terminal domain and a C-terminal domain. It binds the viral genome in a beads-on-a-string conformation and forms a shell or capsid around the enclosed RNA [2,23]. Its identified RNA substrates include transcription regulatory sequences and genomic packaging signals [24,25]. The N protein is heavily phosphorylated which has been shown to enhance the binding affinity of the protein to the viral RNA as opposed to the nonviral RNA [26]. The N protein can also bind the M protein during the assembly of new viral particles and enhance the efficiency of the assembly process [27].

The M protein is a 25–30 kDa protein with three transmembrane domains. It has a small N-terminal glycosylated ectodomain and a large C-terminal endodomain that extends 6–8 nm into the viral particle where it comes into contact with the nucleocapsid [28,29]. M protein exists as a dimer and it may adopt two different conformations. The conformational changes between elongated M proteins and compact M proteins affect rigidity or flexibility for the viral membrane and membrane curvature. In addition, it allows the M protein to bind to the N protein in an efficient manner [17]. The M protein functions mainly in the assembly of new viral particles, where host factors and viral components are put together to form new viruses [30].

The E protein is an 8–12 kDa transmembrane protein found in small quantities within the virus. It is actively synthesized during viral infection localizing at the ER-Golgi intermediate compartment (ERGIC) of the cell, where viral budding and morphogenesis take place [18]. It has an N-terminal ectodomain and a C-terminal endodomain. It has ion channel activity which plays a significant role in viral pathogenesis and virus-host interaction [18].

The S protein is a Class I fusion glycoprotein of about 150 kDa. It consists of a large ectodomain, a single transmembrane anchor and a very small endodomain forming an intracellular tail [31]. The ectodomain, in turn, consists of a receptor binding unit called the S1 subunit and a membrane fusion unit called the S2 subunit. For CoV to enter a host cell, the S1 subunit binds to a specific receptor on the surface of a host cell. Consequently, the S2 subunit fuses the viral membrane and the host cell membrane together [31]. This fusion permits the entry of the viral genome into the host cell. Different CoVs use different cell surface receptors to enter into host cells. When SARS-CoV-2 enters host cells, the S protein of SARS-CoV-2 binds to angiotensin converting enzyme 2 (ACE2), a type I membrane protein expressed in lungs, heart, kidneys, and intestine, to enter host cells [32,33,34,35]. In this review, we demonstrate the entering mechanism of SARS-CoV-2 in host cells and small molecule inhibitors of SARS-CoV-2 cell-entry.

## 3. Entering Mechanism of SARS-CoV-2 and the Role of Its Spike Protein

**Figure 2:**
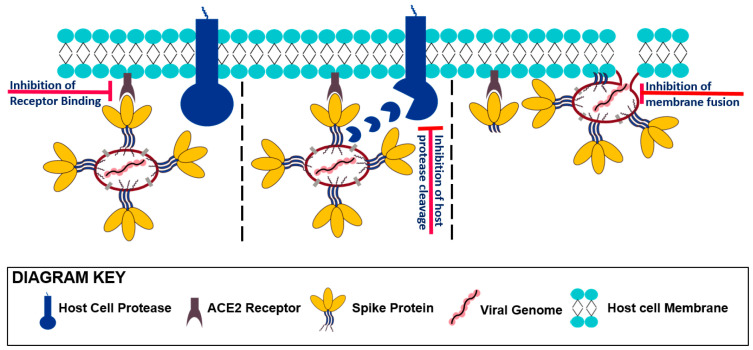
Entry mechanism of Sars-CoV-2 into human host cells. SARS-CoV-2 enters host cell by attaching its surface-anchored spike protein to the cell surface receptor ACE2. Such attachment activates various host cell proteases and causes them to cleave the spike protein at a site near the S1/S2 boundary. Cleavage of the spike protein, in turn, facilitates viral fusion and subsequent insertion of the viral genome into the host cell. Sites of inhibition by small molecules, namely the site of receptor binding, the site of host protein cleavage and the site of membrane fusion, are marked at each stage of viral entry. The schematic is not drawn to scale. Enlargement has been employed to better depict the viral entry mechanism.

Understanding the entry mechanism of the SARS-CoV-2 into human cells is vital for determining its infectivity and pathogenesis as well as for designing targeted therapies against it. Generally, SARS-CoV-2 enters host cells by attaching its surface-anchored spike protein to a receptor (ACE2) on the surface of host cells as illustrated in Figure 2. Such attachment activates various host cell proteases and causes them to cleave the spike protein at a site near the S1/S2 boundary. Cleavage of the spike protein, in turn, facilitates viral fusion and subsequent entrance into the host cell [36].

The spike protein of SARS-CoV-2 is made up of 1273 amino acids and is divided into the S1 subunit, residues 13–685, and S2 subunit, residues 686–1273. The S1 subunit of SARS-CoV-2 contains a receptor binding domain (RBD), residues 319–541, which subsequently includes the receptor binding motif (RBM) that binds with specific residues in the ACE2 extracellular peptidase domain to allow for viral attachment. The S2 subunit mediates viral fusion upon proteolytic cleavage by host cell proteases and contains sub regions including the fusion peptide (FP), heptad repeat 1 (HR1), heptad repeat 2 (HR2), transmembrane domain (TM) and cytoplasmic domain fusion (CP) [37].

### 3.1. Receptor Recognition and Binding

As mentioned previously, the attachment of SARS-CoV-2 to the cell membrane is mediated by the interaction of its trimeric spike protein with the host receptor ACE2, the same host receptor used by SARS-CoV to enter cells [38,39,40].

To uncover the detailed molecular interaction of the SARS-CoV-2 spike RBD with the peptidase domain of ACE2, studies that determined the structures of SARS-CoV-2 spike RBD−ACE2 binding interface were reviewed. The published structures were resolved using either X-ray crystallography or cryo-electron microscopy (cryo-EM) techniques [37,41,42,43]. Using the cryo-EM technique, one group resolved a ternary complex (PDB: 6M17) consisting of the RBD of the SARS-CoV-2 spike in contact with ACE2-B0AT1 complex, where B0AT1 is an amino acid membrane-trafficking transporter [37]. Next, other groups determined two crystal structures of the SARS-CoV-2 RBD in complex with ACE2 (PDB: 6M0J and PDB: 6VW1) [42,43]. Later, one more group resolved the crystal structure of the C-terminal domain of SARS-CoV-2 spike bound to ACE2 (PDB: 6LZG) [41].

**Figure 3:**
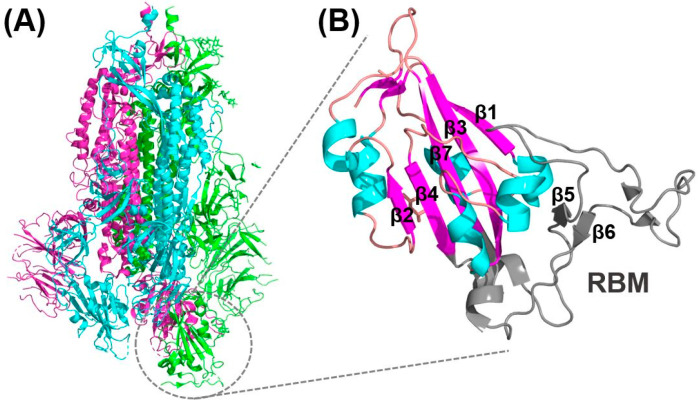
SARS-CoV-2 spike protein. (**A**) The 3D structure of the SARS-CoV-2 trimeric spike protein colored by its monomers. (**B**) Magnified visualization of the secondary structure of the SARS-CoV-2 spike RBD where Beta sheets are labelled and colored in magenta, alpha helices are colored in cyan and loops are colored in pink. The RBM which directly interacts with ACE2 is colored in gray and its beta sheets are labelled. This figure was generated using PyMOL (Version 2) from the PDB: 6VSB for (**A**) and PDB: 6M0J for (**B**).

As observed from the resolved structures of SARS-CoV-2/ACE2 interfaces, the RBD adopts a secondary structure consisting of five twisted antiparallel β-sheets that are connected with short alpha-helices and loops [36,37,41,43]. The receptor binding motif (RBM), which has the major ACE2-interacting residues, is an extended region that runs between β4 and β7 and has the short β5 and β6 strands, α4 and α5 helices and loops as can be seen in Figure 3. The RBM forms the majority of the polar interactions with the alpha-1 helix of ACE2 peptidase domain [36,37,41,43]. The RBD contains 9 cysteine residues which form four disulfide bonds that contribute to the stability of the RBD. The resolved structures indicate that each peptidase domain of ACE2 can accommodate one RBD of a SARS-CoV-2 spike protein.

**Figure 4:**
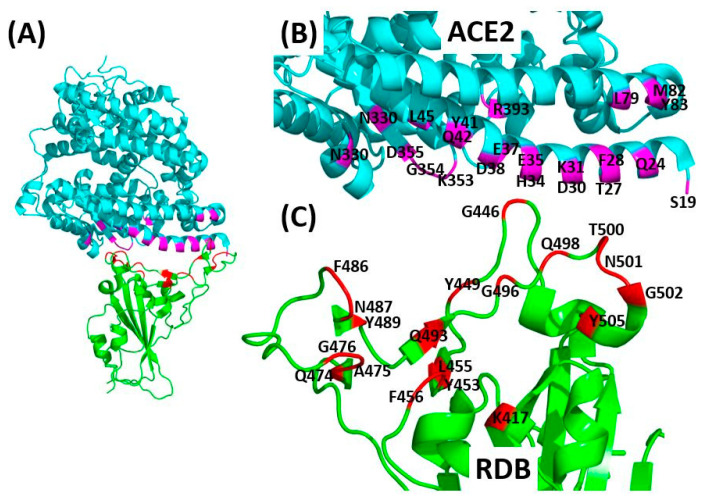
SARS CoV-2 spike RBD−ACE2 binding interface. (**A**) The resolved 3D crystal structure of the SARS CoV-2 spike RBD in contact with human ACE2 receptor (PDB: 6LZG). The ACE2 is colored in cyan and its interacting amino acids are colored in magenta while the RBD is colored in green and its interacting amino acids are colored in red. (**B**) Zoom-in (or enlarged) view of the ACE2 showing the residues that are involved in hydrogen binding with the RBD. (**C**) Zoom-in view of the RBD showing the residues that are involved in hydrogen binding with ACE2. The labelled amino acids of ACE2 and RBD in (**B**,**C**) refer to all the interacting residues that are reported in the four resolved RBD−ACE2 structures (PDBs 6VW1, 6M0J, 6M17 and 6LZG); PDB 6LZG was used as a representative only. Refer to Table 1 for the detailed description of hydrogen bonds formed by the highlighted residues. This figure was generated using PyMOL (Version 2).

By reviewing the four PDB structures, it is observed that the RBD−ACE2 binding interface is stabilized by a high number of hydrophilic or polar interactions between RBD and ACE2 residues. Each of the four studies has reported different interacting RBD and ACE2 residues that are involved in stabilizing the interface through hydrogen binding [36,37,41,43]. Figure 4 represents one of the four resolved structures of SARS CoV-2 spike RBD−ACE2 interfaces and displays all RBD and ACE2 residues that were reported in [36,37,41,43] to form hydrogen bonds. Specifically, the interacting RBD amino acids include K417, G446, Y449, Y453, L455, F456, Q474, A475, G476, F486, N487, Y489, Q493, G496, Q498, T500, N501, G502 and Y505. As for ACE2, its interacting residues are within the extracellular peptidase domain (amino acids 19–615), including S19, Q24, T27, F28, D30, K31, H34, E35, E37, D38, Y41, Q42, L45, L79, M82, Y83, N330, K353, G354, D355, R357 and R393. Table 1 displays the detailed interacting RBD and ACE2 residues for each of the four resolved PDB structures as described in [36,37,41,43].

**Figure 5:**
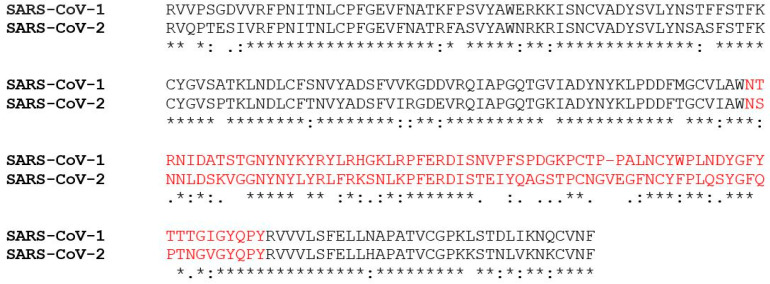
Pairwise amino acid sequence alignment of the receptor binding domain (RBD) of SARS-CoV-1 (Uniprot ID: P59594) and SARS-CoV-2 (Uniprot ID: P0DTC2) performed using Clustal Omega [44]. The asterisk * denotes positions with a single, fully conserved residue. The colon: denotes positions with conservation of groups of strongly similar properties. The period denotes conservation of groups of weakly similar properties. The region labelled in red denotes the receptor binding motif (RBM). RBD of SARS-CoV-1 and SARS-CoV-2 share a 73% identity while their RBMs share only a 50% identity. The amino acid substitutions in the RBM of SARS-CoV-2 result in a greater number of residues interacting with the host cell surface receptor.

All studies that have determined the binding interfaces of SARS-CoV-2 spike with ACE2 found that the RBD of SARS-CoV-2 and SARS-CoV are closely related where the essential residues of both RBDs are conserved or highly similar. Figure 5 demonstrates the pairwise sequence alignment of the RBDs of SARS-CoV and SARS-CoV-2 which have a similarity of 73% [44]. However, some structural variations arise from the amino acid substitutions that exist in the RBM of SARS-CoV and SARS-CoV-2 where their identity is estimated to be only 50%. As reported in [42], the RBM of SARS-CoV-2 (PDB: 6VW1) has a larger interface than that of SARS-CoV with a higher number of interacting residues that form highly stable polar contacts with ACE2, an observation that may explain the higher binding affinity of SARS-CoV-2 to ACE2 that was experimentally measured by some studies [36,42,45]. In fact, different studies have investigated the ACE2 binding affinity of the entire spike or its RBD and showed that the RBD of SARS-CoV-2 has a higher binding affinity to human ACE2 than the RBD of SARS-CoV, while the ACE2 binding affinity of the entire SARS-CoV-2 spike is lower than that of SARS-Cov [36,41,42,45]. This result can be explained by examining the dynamics of the RBD of each of the SARS-CoV and the SARS-CoV-2 spikes. The RBD can adopt either a standing-up or lying-down position where the latter conformation enables the spike protein to avoid immune responses and the former conformation mediates receptor recognition and binding [36,39,45]. Interestingly, the RBD of SARS-CoV-2 is found mostly in the lying-down position in contrast to the RBD of the SARS-CoV spike that predominantly adopts the standing-up position, an observation that may explain the differences in binding affinities of the entire spike as opposed to the RBD of SARS-CoV-2 and SARS-CoV [36,39,45].

### 3.2. Protease Activation/Proteolytic Processing

To allow for viral fusion with the host cell membrane, the SARS-CoV-2 spike undergoes multiple proteolytic cleavage events, performed by host cell proteases, at the boundary between its S1 and S2 subunits and within its S2 subunit [39]. The SARS-CoV-2 spike was found to have a proprotein convertase (PPC) cleavage site at the S1 and S2 boundary that is critical for its entry into the host cell [36].

It was experimentally proven that the human type I transmembrane protein furin is the PPC that leads to the cleavage of S1 and S2 subunits of SARS-CoV-2 spike (residues: R682-R683-A684-R685) [36,39]. The presence of the furin cleavage site at the boundary of the S1 and S2 subunits is specific to the spike of SARS-CoV-2 which is not found to exist in SARS-CoV or any other SARS-related CoVs [36,39,46].

In addition, a plasma-membrane serine protease referred to as transmembrane protease serine-2 (TMPRSS2 protein), is involved in the cleavage and activation of the spike protein of both SARS-CoV-2 and SARS-CoV, to facilitate viral entry into the cell [35,36,47]. The third class of proteases that process the spike protein of SARS-CoV-2 are cathepsins, which are typically found in lysosomes and endosomes. cathepsin-B, a cysteine protease, cleaves spike protein of SARS-CoV-2 but not SARS-CoV whereas cathepsin-L, a cysteine protease, can cleave spikes of both SARS-CoV and SARS-CoV-2 but with higher efficiency towards SARS-CoV [48]. It was then suggested that the collective effect of TMPRSS2 protein and cathepsins are needed in addition to furin to activate the SARS-CoV-2 cell entry [36].

### 3.3. Viral Fusion

Upon the ACE2 recognition by the RBD of SARS-CoV-2 spike and the priming by host cell proteases, the prefusion trimeric spike undergoes several structural rearrangements including the dissociation of the S1 subunit and the transition of S2 subunit to a postfusion conformation in order to allow the viral genome entry into the host cell. The postfusion S2 domain, which facilitates fusion of viral and host cell membranes, is characterized by the formation of a 6-helix bundle that results from the interaction between two heptad repeats, HR1 and HR2. In addition, the S2 domain contains the fusion peptide region that interacts with the host cell membrane upon activation by receptor recognition or protease cleavage [35,49,50].

## 4. Small Molecule Inhibitors of SARS-CoV-2 Host Cell Entry

To date, no specific vaccines or drugs have been approved for the treatment of Covid-19. Since the development of an effective vaccine is time consuming, many studies are investigating candidate drugs to combat SARS-CoV-2 infection. Specifically targeting the viral entry process with inhibitory molecules is considered one of the main preventative therapies as it could interfere with the viral infection at an early point in time. Small molecules can either inhibit viral attachment, priming by the host’s proteases or viral fusion. This section reviews studies that have reported the inhibition of SARS-CoV-2 entry pathways by the targeting of SARS-CoV-2 spike protein, human ACE2 receptor and the different host cell proteases that activate viral fusion. This review focuses on the use of small molecule inhibitors rather than antibodies and peptides due to the well-known advantages of small molecules, such as their high stability in biological fluids and their relatively high immunological tolerance [51]. In addition, the use of small molecule drugs is one of the main therapeutic strategies that is continuously under development and implementation in various viral models including Hepatitis B Virus, Severe Acute Respiratory Syndrome Coronavirus, influenza, Ebola, Zika, Hendra, and Nipah Viruses [52,53,54,55].

Studies that have identified small molecule inhibitors of the SARS-CoV-2 spike by experimental validation and computational prediction are listed in Table 2 and Table 3, respectively. Additionally, studies that have identified small molecule inhibitors of human ACE2 receptors or the host cell proteases by experimental validation and computational prediction are listed in Table 4 and Table 5, respectively. Given the recent emergence of Covid-19, it is important to note that the majority of the studies have only performed computational docking analyses to identify small molecule inhibitors of different SARS-CoV-2 entry targets; however, we believe that these studies should be considered for in vitro future tests to validate the potential therapeutic applications of their computationally proposed small molecules. Specifically, the computationally proposed small molecules with low docking scores can be further screened through in vitro cell-based assays, such as cell−cell and cell−viral fusion methods, to validate their efficacy in preventing viral entry [56].

As can be noticed in Table 2, Table 3, Table 4 and Table 5, a wide range of small molecules are proposed as inhibitors of SARS-CoV-2 entry. Some of these small molecules are clinically approved drugs for the treatment of other diseases which may suggest repurposing them for the treatment of Covid-19 patients. However, small molecules which are classified as investigational drugs may require further validation to assess their efficacy and safety in treating Covid-19 or any other disorder. In addition, many of the computationally-proposed SARS-CoV-2 entry inhibitors are natural small molecules including flavonoids. This class of small molecules are recognized as nutraceuticals, or nutritional supplements, that are clinically-safe and can be administrated to ensure continuous protection against SARS-CoV-2 infection if experimentally proven to interfere with one of the SARS-CoV-2 entry targets. 

### 4.1. SARS-CoV-2 Spike Protein as the Drug Target

#### 4.1.1. Small Molecule Inhibitors of Viral Attachment (RBD/ACE2 Interface)

Using a computational docking approach, Wu et al. screened databases of approved drugs including antiviral and natural compounds against different SARS-CoV-2 targets, one of which is the structural spike protein [63]. Results show that multiple drugs, such as anti-hypertensive, anticoagulant, antibacterial and antifungal, and some natural flavonoids have strong binding affinities with the spike protein (Table 3). However, only hesperidin, a bioflavonoid, binds with the RBD of the spike protein that recognizes ACE2, which in turn could interfere with the viral receptor recognition step [63]. The other docking-predicted compounds did not bind the RBD of the spike protein and it is not reported which residues of the spike protein are involved in the drug binding interfaces [63]. Another study by Wahedi et al. found that resveratrol, a natural polyphenol, is able to bind with low binding energy to the complex formed by SARS-CoV-2 RBD−ACE2 (Table 3) [65].

Using small molecules to block the interaction of SARS-CoV-2 spike RBD with ACE2 has not yet been experimentally tested. However, since SARS-CoV-2 and SARS-CoV spikes share a high structural similarity, the small molecules that were identified to block the RBD of the SARS-CoV spike can be validated for SARS-CoV-2. Specifically, the small molecule, Emodin which is an anthraquinone derived from a Chinese medicinal herb, blocked the interaction of spike RBD with ACE2 in a dose-dependent manner [77]. In addition, oxazole-carboxamide derivative, SSAA09E2, was shown to bind to SARS-CoV RBD and interfere with its recognition of ACE2 [78].

#### 4.1.2. Small Molecule Inhibitors of Viral Fusion (Spike S2 Subdomain)

In an attempt to discover viral fusion inhibitors that target the S2 domain of the SARS-CoV-2 spike, a computational-based study adopted a drug repurposing approach to identify candidate inhibitors that could bind the spike HR1 internal region (residues 897−920) to prevent its transition along with HR2 to the 6-helix stabilized postfusion structure [50]. The study scored the top-ten ranked small molecule drugs that showed high binding to the HR1 central cavity of the spike protein while taking into account their docking scores, clinical trial reports, antiviral, physiological and side effects (Table 3) [50].

Since the spike protein is considered an attractive drug target, a recent study has employed molecular dynamics to analyze the evolutionary variability of the SARS-CoV-2 spike against 791 viral genome sequences [66]. The S2 subunit was found to be the least variable region: it forms a novel binding site or a cavity from the three spike monomers that could be used as a drug target. The novel trimeric binding cavity was screened across a library of FDA approved drugs where the top scoring compounds, including Chitosan, Rapamycin, Paclitaxel, Everolimus, Ritonavir, SelaMeerin and Danoprevir, are suggested as anti-SARS-CoV-2 drugs (Table 3) [66].

Another approved antiviral and anti-influenza drug, arbidol, has been linked to lower infection rates of SARS-CoV-2 [79]. In an in vitro study, arbidol was shown to efficiently inhibit viral entry into cells by significantly reducing the binding of SARS-CoV-2 spike to the cells as well as by preventing viral release from intracellular vesicles [57]. A molecular dynamics analysis has revealed that arbidol impedes the S2 trimerization of SARS-CoV-2 spike [58].

Furthermore, an anti-inflammatory natural drug, cepharanthine showed entry and postentry inhibition potencies against SARS-CoV-2 as revealed by a study that used 2019-nCoVr as a model virus for SARS-CoV-2. The 2019-nCoVr is 92% homologous to SARS-CoV-2 [59].

Based on preliminary clinical data, chloroquine and hydroxychloroquine, the approved drugs for the treatment of malaria, showed potential therapeutic results in in vitro models and in the treatment of Covid-19 patients [60,61,80,81,82]. Due to the known endocytosis inhibition of chloroquine and hydroxychloroquine, it was suggested that they display their antiviral effect by means of preventing the pH-dependent fusion/replication of SARS-CoV-2; but their exact targets are not yet known [60]. However, two studies have reported the irresponsive effects upon treatment of chloroquine and hydroxychloroquine in in vitro models which raises concerns about their use in treating Covid-19 patients [62,83]. Recently, various clinical studies have investigated the effectiveness of hydroxychloroquine for the prevention and/or treatment of SARS-CoV-2 infection to provide robust support regarding its clinical use in Covid-19 patients. The reported result show low clinical benefits of this drug for either preventing the transmission of SARS-CoV-2, treating mild to moderate Covid-19 patients or reducing incidences of intubation or death of hospitalized Covid-19 patients [84,85,86,87,88,89,90].

Small molecule fusion inhibitors that have been previously identified to bind the SARS-CoV spike can be also tested for fusion inhibition of SARS-CoV-2. A benzamide derivative compound, SSAA09E3, was identified as a SARS-CoV entry inhibitor possibly by blocking the fusion step, however, its exact target is not clear yet [78]. In addition, three small molecules, tetra-O-galloyl-D-glucose (TGG), luteolin, and Quercetin, can bind the S2 subunit and block SARS-CoV fusion and cell entry [59].

### 4.2. Host Cell Receptor and Proteases as the Drug Targets

Another viral entry inhibition approach includes inhibiting host cell components such as the host cell receptor, ACE2, to prevent the cellular attachment of the spike protein or inhibiting the host proteases that are required for priming and activation of SARS-CoV-2 spike to allow for its fusion upon receptor recognition. However, this approach requires more attention as interfering with the host’s targets might be linked with unwanted substantial side effects.

#### 4.2.1. Protease Small Molecule Inhibitors

One of the potential therapeutic strategies that is currently under investigation is the inhibition of host cell proteases, such as TMPRSS2, furin and cathepsins that are required for priming and activation of the SARS-CoV-2 spike. The inhibitor of the host cell protease TMPRSS2, camostat mesylate, is a clinically approved serine protease inhibitor that was shown to prevent SARS-CoV-2 infection in human lung cells in in vitro tests and is now under clinical trial [35]. In addition, another potent inhibitor of TMPRSS2, nafamostat mesylate, was shown to prevent SARS-CoV-2 entry into human lung cells with higher efficiency as compared to camostat mesylate and when combined with heparin, it can target coagulopathy in severe Covid-19 cases (Table 4) [67,68]. Interestingly, the well-known antiviral drug, remdesivir, which is an inhibitor of the viral RNA-dependent RNA polymerase, showed a low computational docking score with TMPRSS2 [63].

Unlike TMPRSS2, furin has various physiological roles as a serine protease and its inhibition to block SARS-CoV-2 activation should be limited to a short well-tolerated period. One study had successfully used furin inhibitors, decanoyl-RVKR-chloromethylketone (CMK), a short peptide inhibitor, and naphthofluorescein, to block furin cleavage of the SARS-CoV-2 spike (Table 4) [69,91].

Another host cell protease that could be targeted against SARS-CoV-2 entry is the Cathepsin-L that promotes viral fusion in the cell endosomes and the subsequent RNA release and replication. A recent review, published in late May, 2020, has evaluated seven Cathepsin-L inhibitors that have previously shown anticoronavirus effects as listed in Table 4 [70]. However, prolonged use of cathepsins inhibitors is not recommended as the long-term inhibition of Cathepsin-L might lead to tissue fibrosis or pulmonary fibrosis when used to treat lung injuries resulting from SARS-CoV-2 infection [70].

A combination of serine and cysteine protease inhibitors can also be used for preventing viral entry by blocking both the endosomal and nonendosomal pathways [92].

#### 4.2.2. ACE2 Small Molecule Inhibitors

Targeting ACE2 to inhibit viral entry is one therapeutic strategy to combat Covid-19 as the ACE2 receptor is the main receptor recognized by the SARS-CoV-2 spike. However, inhibiting ACE2 needs to be tightly regulated as ACE2 is part of the renin-angiotensin-aldosterone (RAAS) system and is involved in maintaining cardiac function by producing the vasodilator peptide angiotensin 1–7 from angiotensin II [93].

On the basis of structure-based screening of a large number of small molecules against the ACE2 active site, an early study has shown that *N*-(2-aminoethyl)-1 aziridine-ethanamine or NAAE can inhibit ACE2 catalytic function and prevent SARS-CoV infection in an in vitro model (Table 4) [72]. Another potent small molecule catalytic inhibitor of ACE2 is (S,S)-2-[1-carboxy-2-[3-(3,5-dichlorobenzyl)-3*H*-imidazol4-yl]-ethylamino]-4-methylpentanoic acid which was resolved bound to ACE2 using X-ray crystallography (PDB: 1R4L) [73]. Another ACE2 inhibitor small molecule is the natural metal chelator, Nicotianamine or *N*-[*N*-(3-ami-no-3-carboxypropyl)-3-amino-3-carboxypropyl]-azetidine-2-carboxylic acid which is isolated from soybean [74].

More recently, a study has conducted computational-based approaches to identify new small molecule inhibitors of the ACE2 active sites which are believed to induce a conformational change in the ACE2 N-terminal region that bind SARS-CoV-2 spike RBD to prevent viral attachment [75]. The top-ranked small molecule drugs which showed strong binding to the ACE2 active sites are lividomycin, burixafor, quisinostat, fluprofylline, pemetrexed, spirofylline, edotecarin, and diniprofylline (Table 5) [75]. Another study employed molecular docking of around 500,000 compounds to identify 20 potent inhibitors that bind the ACE2 spike-binding residues (Table 5) [76]. Additionally, the previously mentioned study, by Wu et.al, also performed molecular docking against ACE2 and revealed several small molecules that could bind to ACE2 with low energy, however, the predicted small molecules do not specifically target the ACE2 residues that bind to RBD (Table 5) [63].

Again, the use of ACE2 inhibitors should be tightly regulated due to the critical role of ACE2 in cardiac physiology. In fact, serious concerns have been raised over whether drugs that act on the RAAS system can worsen or alleviate Covid-19 infection, especially in relation to cardiac and hypertensive patients. Some studies claim that ACE inhibitors and angiotensin receptor blockers worsen Covid-19 infection by increasing the expression of ACE2 which increases the chance of viral entry into the organs of the body [94,95]. On the other hand, other studies claim beneficial effects of ACE inhibitors and angiotensin receptor blockers in the treatment of Covid-19 and argue that the increase in ACE2 expression and the corresponding deviation of angiotensin I to angiotensin 1–9 and 1–7 exerts protective pulmonary and cardiac effects which protect patients from acute lung damage during SARS-CoV-2 infection [96,97].

## 5. Conclusions

The recent outbreak of SARS-CoV-2 has raised serious global concerns as the number of infections are increasing every day. Research groups around the world are trying to identify and develop effective treatments against Covid-19. However, to date, there are no specific treatments approved for this disease. Viral entry constitutes the first step of viral infection and represents an attractive intervention step. In this review, we summarized literature on small molecules experimentally shown or computationally predicted to inhibit SARS-CoV-2 entry into host cells. Such small molecules interfere with the viral entry process at one of three focal stages: either at receptor recognition and binding stage or at protease processing stage or at fusion stage. We believe that this review serves as a guide for future in vitro and in vivo validation studies to identify effective SARS-CoV-2 entry inhibitors.

## Figures and Tables

**Table 1:** The hydrogen bonds formed at the SARS CoV-2 RBD–ACE2 interfaces of PDBs 6VW1, 6M0J, 6M17 and 6LZG.

SARS CoV-2-RBD Residues	K417	G446	Y449	Y453	L455	F456	Q474	A475	G476	F486	N487	Y489	Q493	G496	Q498	T500	N501	G502	Y505
PDB: 6M17 [37]																			
ACE2 residues	D30	-	-	H34	-	-	Q24	-	-	-	-	-	-	-	Q42	R357	Y41 K353 R357	-	-
PDB: 6VW1 [42]																			
ACE2 residues	-	-	D38	H34	H34	T27 K31	-	S19 T27	Q24	L79 M82 Y83	Q24 Y83	T27 F28	K31 E35	K353	Y41 Q42 L45	Y41 N330 D355 R357	Y41 K353	K353 G354	E37 K353 G354
PDB: 6M0J [43]																			
ACE2 residues	D30	Q42	D38 Q42	-	-	-	-	-	-	-	Q24 Y83	Y83	E35	-	-	Y41	Y41	K353	E37 R393
PDB: 6LZG [41]																			
ACE2 residues	D30	Q42	D38 Q42	H34	-	-	-	S19	-	-	Q24 Y83	-	-	K353	Q42	Y41 D355	Y41	K353	-

**Table 2:** Small molecules identified through experimental approaches for targeting the SARS-CoV-2 spike protein.

Small Molecule Name	Structure	Type	Target	IC50/EC50	Ref.
Arbidol	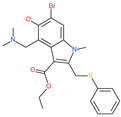	Anti-influenza virus drug	Viral attachment and entry and targeting the trimerization of SARS-CoV-2 spike	4.11 μM	[57,58]
Cepharanthine (CEP)	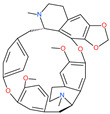	A naturally occurring anti-inflammatory and anti-neoplastic alkaloid drug	Inhibits entry and post-entry of a SARS-CoV-2 homologous viral model (92% homology)	0.98 μM	[59]
Chloroquine	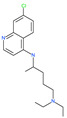	Approved malaria drug	Viral fusion through endocytosis	1.13 μM	[60,61]
Hydroxychloroquine	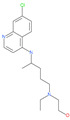	Approved malaria drug	Viral fusion through endocytosis	0.72 μM	[62]

**Table 3:** Small molecules identified through computational approaches for targeting SARS-CoV-2 spike protein.

Small Molecule Name	Structure	Type	Target	Docking Score	Ref.
Hesperidin	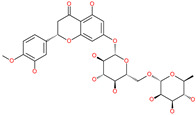	Bioflavonoid	Spike RBD and ACE2	−20.63 kcal/mol (RBD) −3.12 kcal/mol (ACE2)	[63,64]
Rescinnamine	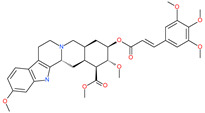	Anti-hypertensive drug	Spike protein (not RBD)	−35.31 kcal/mol	[63]
Iloprost	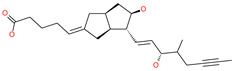	Anti-hypertensive drug	Spike protein (not RBD)	−26.19 kcal/mol	[63]
Prazosin	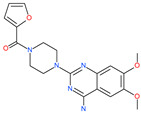	Anti-hypertensive drug	Spike protein (not RBD)	−19.78 kcal/mol	[63]
Posaconazole	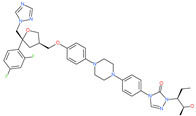	Antifungal drug	Spike protein (not RBD)	−33.04 kcal/mol	[63]
Itraconazole	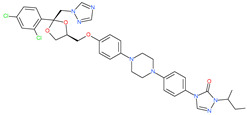	Antifungal drug	Spike protein (not RBD)	−31.03 kcal/mol	[63]
Sulfasalazine	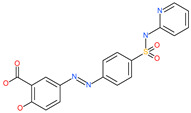	Antibacterial drug	Spike protein (not RBD)	−22.21 kcal/mol	[63]
Azlocillin	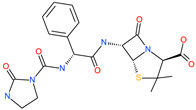	Antibacterial drug	Spike protein (not RBD)	−36.64 kcal/mol	[63]
Penicillin	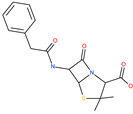	Antibacterial drug	Spike protein (not RBD)	−35.85 kcal/mol	[63]
Cefsulodin	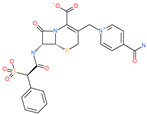	Antibacterial drug	Spike protein (not RBD)	−35.25 kcal/mol	[63]
Dabigatran etexilate	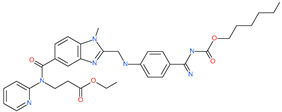	Anticoagulant drug	Spike protein (not RBD)	−27.41 kcal/mol	[63]
Licoflavonol	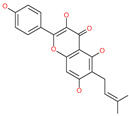	Natural flavonoid	Spike protein (not RBD)	−12.67 kcal/mol	[63]
Cosmosiin	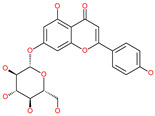	Natural flavonoid	Spike protein (not RBD)	−17.54 kcal/mol	[63]
Neohesperidin	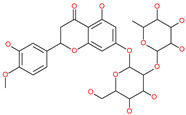	Natural flavonoid	Spike protein (not RBD)	−24.75 kcal/mol	[63]
Mangostin	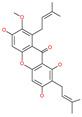	Natural flavonoid	Spike protein (not RBD)	−22.77 kcal/mol	[63]
Kouitchenside D	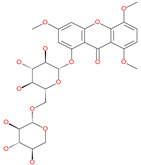	Natural flavonoid	Spike protein (not RBD)	−9.75 kcal/mol	[63]
Excoecariatoxin	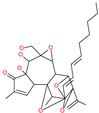	Natural flavonoid	Spike protein (not RBD)	−17.8 kcal/mol	[63]
Phyllaemblicin G7	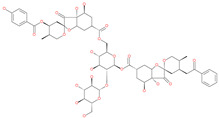	Natural flavonoid	Spike protein (not RBD)	−12.07 kcal/mol (Spike)	[63]
Piceatannol	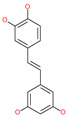	Natural flavonoid	Spike protein (both RBD and non-RBD]	−27.58 kcal/mol	[63,65]
Resveratrol	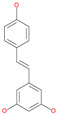	Natural polyphenol	ACE2-RBD complex (PDB: 6VW1)	−8.0 kcal/mol	[65]
31h-phthalocyanine	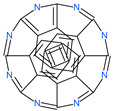	Investigational anticancer and antiviral drug	HR1 central cavity of Spike protein	−16.3 kcal/mol	[50]
Hypericin	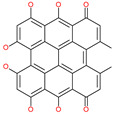	Investigational anticancer and antiviral drug	HR1 central cavity of Spike protein	−15.1 kcal/mol	[50]
Ergotamine	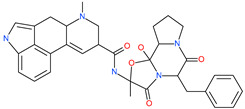	Approved drug for migraine	HR1 central cavity of Spike protein	−13.2 kcal/mol	[50]
TMC-647055	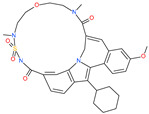	Investigational antiviral drug	HR1 central cavity of Spike protein	−12.5 kcal/mol	[50]
Quarfloxin	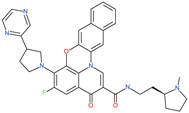	Investigational anticancer drug	HR1 central cavity of Spike protein	−12.6 kcal/mol	[50]
Tepotinib	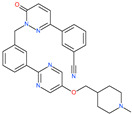	Investigational anticancer drug	HR1 central cavity of Spike protein	−12.0 kcal/mol	[50]
Laniquidar	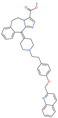	Investigational anticancer drug	HR1 central cavity of Spike protein	−12.8 kcal/mol	[50]
Tadalafil	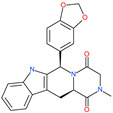	Approved drug for erectile dysfunction and pulmonary arterial hypertension	HR1 central cavity of Spike protein	−12.4 kcal/mol	[50]
JNJ-10311795	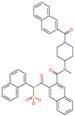	Experimental drug	HR1 central cavity of Spike protein	−13.1 kcal/mol	[50]
TZ2PA6	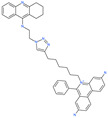	Experimental drug	HR1 central cavity of Spike protein	−12.7 kcal/mol	[50]
Chitosan	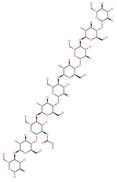	Antibacterial, nontoxic biopolymer	Cavity formed by the three spike S2 subunits	−67.49 kcal/mol	[66]
Rapamycin	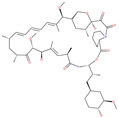	Approved mTOR inhibitor	Cavity formed by the three spike S2 subunits	−49.28 kcal/mol	[66]
Paclitaxel	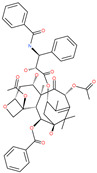	Approved anticancer	Cavity formed by the three spike S2 subunits	−45.84 kcal/mol	[66]
SelaMeerin (Selamectin)	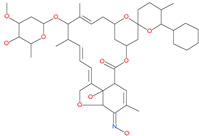	Vet approved antiparasitic	Cavity formed by the three spike S2 subunits	−44.24 kcal/mol	[66]
Everolimus	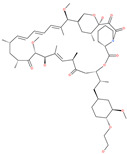	Approved mTOR inhibitor	Cavity formed by the three spike S2 subunits	−41.80 kcal/mol	[66]
Ritonavir	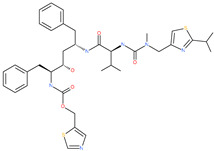	Approved antiviral (HIV protease inhibitor)	Cavity formed by the three spike S2 subunits	−37.92 kcal/mol	[66]
Danoprevir	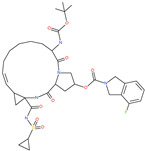	Investigational antiviral drug	Cavity formed by the three spike S2 subunits	−35.09 kcal/mol	[66]

**Table 4:** Small molecules identified through experimental approaches for targeting host cell proteases and ACE2.

Small Molecule Name	Structure	Type	Target	IC50/EC50	Ref.
Camostat mesylate	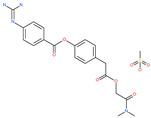	Chronic pancreatitis drug	Host cell protease: TMPRSS2	87 nM	[35,67]
Nafamostat mesylate	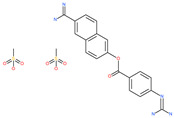	Anticoagulant drug	Host cell protease: TMPRSS2	5 nM	[67,68]
Naphthofluorescein	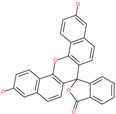	-	Host cell protease: furin	-	[69,70]
EST(23,25)*trans*-epoxysuccinyl-l-leucylamindo-3-methylbutane ethyl ester	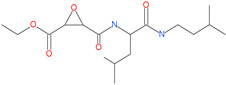	-	Host cell protease: Cathepsin-L and Cathepsin-B	-	[69,70]
Compound K11777	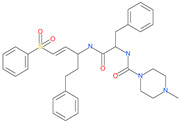	-	Host cell protease: Cathepsin-L	0.68 nM	[69,70]
Compound 5705213	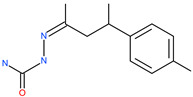	-	Host cell protease: Cathepsin-L	9 uM	[69,70]
Tetrahydroquinoline oxocarbazate	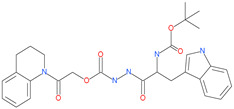	-	Host cell protease: Cathepsin-L	Time-dependent inhibition at IC50 from 6.9 ± 1.0 nM (immediately) to 2.3 ± 0.1 nM (1 h) to 1.2 ± 0.1 nM (2 h) to 0.4 ± 0.1 nM (4 h);	[69,70,71]
Compound SSAA09E1	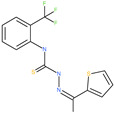	-	Host cell protease: Cathepsin-L	5.22 uM	[69,70]
NAAE	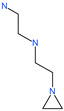	Experimental ACE2 inhibitor	ACE2	−23.7 kcal/mol IC50 of 57 ± 7 μmol/L	[72]
MLN-4760	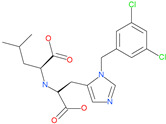	Investigational ACE2 inhibitor	ACE2	0.44 nM	[73]
Nicotianamine	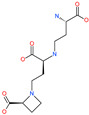	Natural metal chelator	ACE2	84 nM	[74]

**Table 5:** Small molecules identified through computational approaches for targeting host ACE2.

Small Molecule Name	Structure	Type	Target	Docking Score	Ref.
Lividomycin	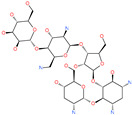	-	ACE2	−2145.79 kcal/mol	[75]
Burixafor	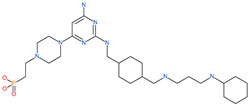	Investigational anticancer drug	ACE2	−2108.82 kcal/mol	[75]
Quisinostat	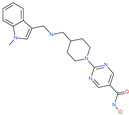	Investigational anticancer drug	ACE2	−1998.77 kcal/mol	[75]
Fluprofylline	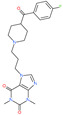	-	ACE2	−1785.00 kcal/mol	[75]
Pemetrexed	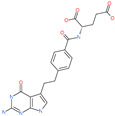	Anticancer drug	ACE2	−1602.58 kcal/mol	[75]
Spirofylline	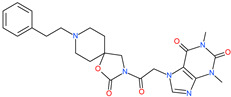	-	ACE2	−1541.73 kcal/mol	[75]
Edotecarin	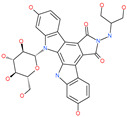	Investigational drug for inhibiting DNA topoisomerase-1	ACE2	−1312.19 kcal/mol	[75]
Diniprofylline	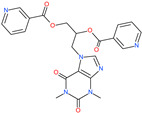	-	ACE2	−1292.42 kcal/mol	[75]
Troglitazone	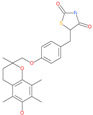	Anti-diabetes drug	ACE2	−21.10 kcal/mol	[63]
Losartan	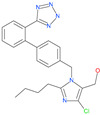	Anti-hypertensive drug	ACE2	−21.49 kcal/mol	[63]
Ergotamine	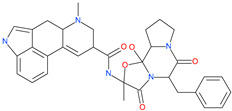	Analgesia drug	ACE2	−14.74 kcal/mol	[63]
Cefmenoxime	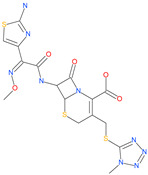	Antibacterial drug	ACE2	−13.49 kcal/mol	[63]
**Silybin**	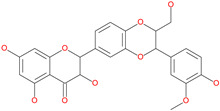	Hepatoprotective drug	ACE2	−25.93 kcal/mol	[63]
Xanthones	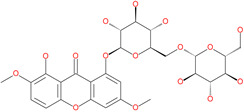	Antiviral and anti-inflammatory effect	ACE2	−29.40 kcal/mol	[63]
*N*′,*N*″-[oxybis(4,1-phenylenecarbonyl)]bis(3-methoxybenzohydrazide)	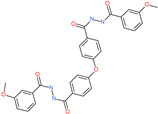	-	ACE2	−5.87 kcal/mol	[76]
2,2′-{1,4-butanediylbis[(4-ethyl-4*H*-1,2,4-triazole-5,3-diyl)thio]}bis(1-phenylethanone)	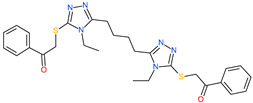	-	ACE2	−5.84 kcal/mol	[76]
*N*,*N*′-bis{4-[(benzylamino)carbonyl]phenyl}malonamide	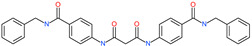	-	ACE2	−5.83 kcal/mol	[76]
*N*,*N*′-[methylenebis(2-hydroxy-4,1-phenylene)]bis[2-(3,4-dimethoxyphenyl)acetamide]	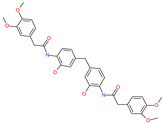	-	ACE2	−5.77 kcal/mol	[76]
ethyl 4-({[(4-allyl-5-{2-[(2,4-dimethylphenyl)amino]-2-oxoethyl}-4*H*-1,2,4-triazol-3-yl)thio]acetyl}amino)benzoate	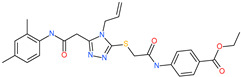	-	ACE2	−5.69 kcal/mol	[76]
2-(4-methoxyphenyl)-*N*-{[4-methyl-5-({2-[(5-methyl-4-phenyl-1,3-thiazol-2-yl)amino]-2-oxoethyl}thio)-4*H*-1,2,4-triazol-3-yl]methyl}acetamide	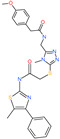	-	ACE2	−5.69 kcal/mol	[76]
*N*,*N*′-1,6-hexanediylbis[2 -(4-isopropylphenoxy)acetamide]	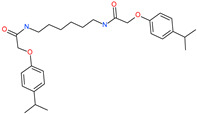	-	ACE2	−5.65 kcal/mol	[76]
*N*′,*N*″-[oxybis(4,1-phenylenecarbonyl)]bis(2-chlorobenzohydrazide)	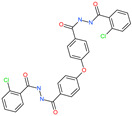	-	ACE2	−5.62 kcal/mol	[76]
*N*-(5-{[2-(dibenzo[b,d]furan-3-ylamino)-2-oxoethyl]thio}-1,3,4-thiadiazol-2-yl)-2-methoxybenzamide	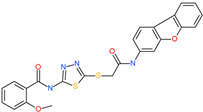	-	ACE2	−5.56 kcal/mol	[76]
*N*,*N*′-1,2-phenylenebis[2-(4-ethylphenoxy)acetamide]	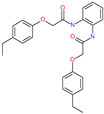	-	ACE2	−5.54 kcal/mol	[76]
ethyl 3-({[(5-{[(3,4-dimethoxybenzoyl)amino]methyl}-4-methyl-4*H*-1,2,4-triazol-3-yl)thio]acetyl}amino)benzoate	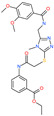	-	ACE2	−5.54 kcal/mol	[76]
*N*,*N*′-(oxydi-4,1-phenylene)bis[2-(2-methoxyphenoxy)acetamide]	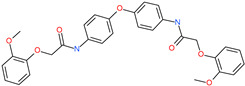	-	ACE2	−5.53 kcal/mol	[76]
*N*,*N*′-4,4′-biphenyldiylbis[2-(2-methoxyphenoxy)acetamide]	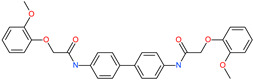	-	ACE2	−5.53 kcal/mol	[76]
1-[(3,4-dimethoxyphenyl)acetyl]-*N*,*N*′-bis(2-thienylmethyl)-1*H*-1,2,4-triazole-3,5-diamine	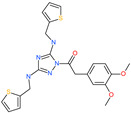	-	ACE2	−5.53 kcal/mol	[76]
*N*,*N*′-1,2-propanediylbis[2-(4-tert-butylphenoxy)acetamide]	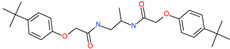	-	ACE2	−5.52 kcal/mol	[76]
4,4′-oxybis[*N*-(2-ethoxyphenyl)benzamide]	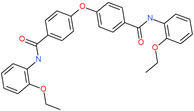	-	ACE2	−5.51 kcal/mol	[76]
2-chloro-*N*-{2-[5-({2-[(3-cyano-4,5,6,7-tetrahydro-1-benzothien-2-yl)amino]-2-oxoethyl}thio)-4-ethyl-4*H*-1,2,4-triazol-3-yl]ethyl}benzamide	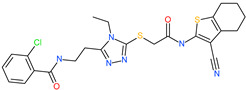	-	ACE2	−5.51 kcal/mol	[76]
*N*-{2-[4-allyl-5-({2-oxo-2-[(4-phenyl-1,3-thiazol-2-yl)amino]ethyl}thio)-4*H*-1,2,4-triazol-3-yl]ethyl}-4-methylbenzamide	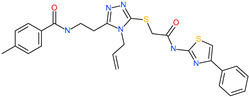	-	ACE2	−5.51 kcal/mol	[76]
*N*-[2-(3,4-dimethoxyphenyl)ethyl]-2-{4-[(isobutylamino)sulfonyl]-2-methylphenoxy}acetamide	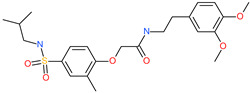	-	ACE2	−5.51 kcal/mol	[76]
*N*~2~-[4-(benzyloxy)phenyl]-*N*~1~-(4-{[(2,6-dimethylphenyl)amino]sulfonyl}phenyl)-*N*~2~-(methylsulfonyl)glycinamide	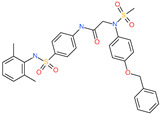	-	ACE2	−5.50 kcal/mol	[76]
Phyllaemblicin G7	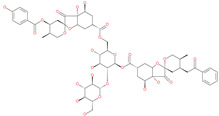	Natural flavonoid	ACE2	−4.33 kcal/mol	[63]
